# Clinical Prediction of Retained Products of Conception: Combining Obstetric History and Ultrasound for Improved Accuracy in Severe Postpartum Hemorrhage

**DOI:** 10.7759/cureus.53651

**Published:** 2024-02-05

**Authors:** Mariko Kurakazu, Masamitsu Kurakazu, Chihiro Kiyoshima, Koichiro Shigekawa, Toyofumi Hirakawa, Kenichi Yoshikawa, Tomohiro Ito, Daichi Urushiyama, Kohei Miyata, Fusanori Yotsumoto

**Affiliations:** 1 Department of Obstetrics and Gynecology, Faculty of Medicine, Fukuoka University, Fukuoka, JPN

**Keywords:** ultrasonography, risk management, placenta, postpartum period, odds ratio, multivariate analysis, hemorrhage, blood transfusion

## Abstract

Background

The current challenge is how to improve the management of postpartum hemorrhage (PPH) to reduce the maternal mortality rate further. This study aimed to investigate whether a combined specific obstetric history and ultrasonographic findings can improve the predictive accuracy of retained products of conception (RPOC) with severe PPH.

Methods

This retrospective study included 56 patients who were diagnosed with RPOC. We extracted the following clinical data: obstetric history of second-trimester miscarriage, the time at which there was clinical suspicion of RPOC after the previous pregnancy (TIME), grayscale ultrasonographic finding (RPOC long-axis length [SIZE]), and color Doppler ultrasonographic finding based on the Gutenberg classification (RPOC hypervascularity). In this study, we defined cases requiring blood transfusion therapy or transcatheter arterial embolization as severe PPH. The patients were divided into two groups according to the presence or absence of severe PPH. The predictors of severe PPH were evaluated using logistic regression models. Model A comprised a combination of second-trimester miscarriage and TIME, Model B comprised a combination of Model A and long-axis SIZE, and Model C comprised a combination of Model B and RPOC hypervascularity.

Results

The multivariable analysis showed that long-axis SIZE was the only significant predictor of severe PPH (odds ratio [OR], 10.38; 95% confidence interval [CI], 2.06-63.86) independent of second-trimester miscarriage, TIME, and RPOC hypervascularity. The c-statistic was higher in Model C (OR, 0.863; 95% CI, 0.731-0.936) than in Model A (OR, 0.723; 95% CI, 0.551-0.847) and Model B (OR, 0.834; 95% CI, 0.677-0.923).

Conclusion

Combining a specific obstetric history (second-trimester miscarriage and TIME) and ultrasonographic findings (long-axis SIZE and RPOC hypervascularity) improves the predictive accuracy of RPOC with severe PPH. This prediction model may be a useful clinical screening tool for RPOC with severe PPH.

## Introduction

Postpartum hemorrhage (PPH) is the leading cause of maternal morbidity and mortality globally [1,2]. In Japan, the maternal mortality rate was 3.5-4.6 per 100,000 live births from 2010 to 2017 [3]. Furthermore, PPH was the most frequent cause of maternal death, accounting for 23% of deaths between 2010 and 2012 [4]. Various exploratory committee investigations on maternal death have been conducted in Japan since 2012. The incidence of PPH progressively decreased from 29% to 7% between 2010 and 2017 [5]. PPH can manifest as primary PPH, which occurs before delivery of the placenta and up to 24 hours after delivery of the fetus and placenta, and secondary PPH, which occurs between 24 hours and six weeks after delivery [6,7]. Primary PPH can be managed successfully. However, improving the management of secondary PPH to reduce the maternal mortality rate further is the current challenge.

Retained products of conception (RPOC) refer to the presence of placental tissue remaining in the uterine cavity after abortion or delivery [7,8]. RPOC is most commonly observed after a second-trimester delivery or termination of pregnancy and is a prevalent cause of secondary PPH [7,9]. However, the appropriate management of RPOC remains unknown because its natural course is unclear [10]. The ability to predict RPOC with severe PPH is particularly important because it guides management strategies that range from conservative treatment to invasive surgery [11-14].

The development of a diagnostic prediction model for RPOC with severe PPH is crucial in clinical practice because it could help determine if a blood transfusion is required. Therefore, this study aimed to investigate whether a combined specific obstetric history and ultrasonographic findings can improve the predictive accuracy of RPOC with severe PPH.

## Materials and methods

Study design and population

This retrospective study was conducted at a university hospital and tertiary perinatal center in Japan. The institutional review board of our institution approved this study (H20-07-011, approved on September 8, 2020), and written informed consent was obtained from all participants.

We included patients who met the following inclusion criteria: patients who were managed for PPH at our institution owing to abortion, miscarriage, or delivery within 12-41 gestational weeks. We excluded cases of atonic hemorrhage, delivery trauma (e.g., cervical laceration, perineal laceration, and vulvar hematoma), placenta accreta, and uterine inversion.

Severe hemorrhage can lead to coagulopathy and further hemorrhage requiring blood transfusions [15,16]. Therefore, we defined cases requiring blood transfusion therapy or transcatheter arterial embolization as severe PPH in this study. We divided the patients into the RPOC with severe PPH group and the RPOC without severe PPH group. We then summarized the patients’ background characteristics and assessed the effects of severe PPH while controlling for potential confounders using logistic regression models.

We extracted the following clinical data from the electronic medical records of our institution: age; parity; height; pre-pregnancy body weight; pre-pregnancy body mass index; assisted reproductive technology (ART); obstetric history including second-trimester miscarriage; delivery modes of the immediate previous pregnancy, such as vaginal delivery, cesarean delivery, spontaneous abortion, and dilatation and curettage (D&C); the time at which there was clinical suspicion of RPOC after the termination of the immediate previous pregnancy (TIME); serum human chorionic gonadotropin (hCG); and hemoglobin (Hb) concentrations at the time of ultrasonography [17,18].

All patients underwent ultrasonography using an ALOKA F37 (Hitachi ALOKA Medical, Tokyo, Japan), Aplio 300 (Canon Medical Systems Corporation, Tochigi, Japan), and Voluson E8 or E10 (GE Healthcare, Tokyo, Japan) ultrasound machine. Transabdominal images were obtained using a transabdominal vector probe at a frequency of 3-6 MHz. Transvaginal images were obtained using a transvaginal vector probe at a frequency of 5-7 MHz. An endometrial mass was defined as an intrauterine mass distinct from the rest of the endometrium [19]. The size (long-axis and short-axis length [SIZE]) of the detected intrauterine mass was recorded. The color Doppler signal was detected and reviewed using the Gutenberg classification for RPOC [13]. Each hyperechogenic avascular mass with the degree of endometrial vascularity was graded as follows: type 1, a mass with minimal vascularity; type 2, a moderately to highly vascularized mass confined to the cavity; and type 3, a highly vascularized mass with highly vascularized myometrium and arterial flow velocity ≥ 100 cm/s [7,13,19].

In ultrasonography with color Doppler, blood that flowed toward the intrauterine mass and was distinct from the rest of the endometrium was defined as RPOC hypervascularity. In addition, the degree of positivity was detected using ultrasonographic vascular flow by type 2 or 3 color Doppler findings based on the Gutenberg classification. Ultrasonographic examinations of all patients were performed by professional obstetrics and gynecology physicians with at least five years of ultrasound experience who were certified as specialists in obstetrics and gynecology by the Japan Society of Obstetrics and Gynecology. Ultrasonographic examinations were performed under the conditions of the special presets for obstetrics and gynecology that were set for each ultrasound model and evaluated after adjustment for optimal images based on the examiner’s judgment.

Statistical analysis

This was a retrospective, observational study. Therefore, no sample size calculations were conducted, and all available participants were included. Continuous variables are summarized as the mean ± standard deviation or median and range, while categorical variables are summarized as the number and percentage. An operating characteristic analysis was conducted to determine the optimal cut-off values for continuous variables [20]. Subsequently, continuous variables were converted to categorical variables. Logistic regression models were used to assess the predictors of RPOC with severe PPH as follows: Model A, specific obstetric histories; Model B, a combination of Model A and grayscale ultrasonographic findings; and Model C, a combination of Model B and color Doppler ultrasonographic findings. The estimated odds ratios (ORs) and their 95% confidence intervals (CIs) were obtained. The discriminatory ability of the statistical models for individuals with RPOC with or without severe PPH was evaluated using the area under the curve. All statistical analyses were conducted using JMP Pro software version 17.0.0 (SAS Institute, Cary, NC). This software automatically deletes cases with missing data and performs a complete set of statistical analyses. Values of p < 0.05 were considered statistically significant.

## Results

**Figure 1:**
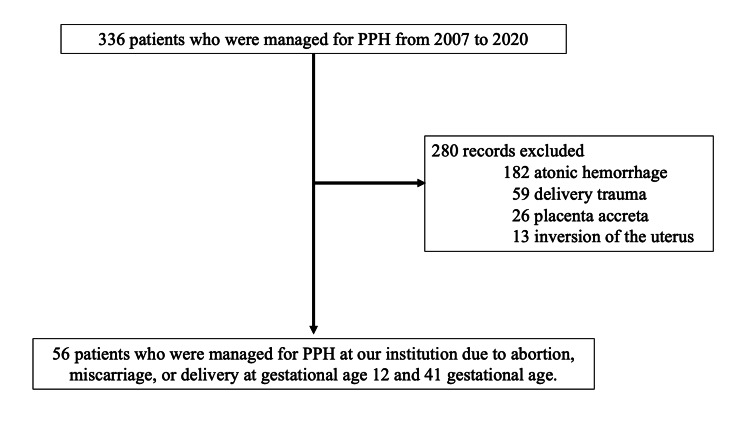
Flow chart of the study population According to the defined exclusion criteria, 56 patients diagnosed with RPOC by ultrasonography and magnetic resonance imaging were enrolled in the present study. RPOC: Retained products of conception; PPH: Postpartum hemorrhage.

Between January 2007 and December 2020, 336 patients met the study's inclusion criteria. After the application of the exclusion criteria, the study population ultimately consisted of 56 patients diagnosed with RPOC by ultrasonography and magnetic resonance imaging (Figure 1). Fourteen patients were classified as RPOC with severe PPH, while the other 42 were classified as RPOC without severe PPH.

The patients’ clinical characteristics are shown in Table 1. Significantly higher values were observed for the following: second-trimester miscarriage (p = 0.0208), TIME (p = 0.0479), long-axis SIZE (p = 0.0103), and RPOC hypervascularity (p = 0.0168) in the RPOC with severe PPH group compared with the RPOC without severe PPH group. TIME and long-axis SIZE were used to assess the cut-off values for the risk factors of RPOC with severe PPH. According to the receiver operating characteristic curve, the cut-off value for TIME was <27 days, while it was >4.4 cm for long-axis SIZE. There was no significant intergroup difference in conception via ART (p = 0.56), RPOC short-axis length (p = 0.28), serum hCG concentrations (p = 0.42), or serum Hb concentrations (p = 0.05).

**Table 1:** Patients’ demographics Values are shown as the median and range or n (%) as appropriate. Hb: Hemoglobin; hCG: Human chorionic gonadotropin; PPH: Postpartum hemorrhage; RPOC: Retained products of conception.

	RPOC with severe PPH group (n = 14)	RPOC without severe PPH group (n = 42)	p
Age (years)	35 (28–43)	34 (20–42)	0.59
Nulliparous	3 (21.4)	18 (42.9)	0.15
Height (cm)	159 (147–167)	160 (146–170)	0.42
Pre-pregnancy body weight (kg)	52.2 (43.5–94.0)	54.6 (40–80.7)	0.60
Pre-pregnancy body mass index (kg/m^2^)	22.5 (18.6–36.7)	21.2 (16.4–34.0)	0.17
Assisted reproductive technology	2 (14.3)	9 (21.4)	0.56
Obstetric history			
Second-trimester miscarriage	5 (35.7)	4 (9.5)	0.0208
Gestational age at the immediate previous pregnancy (weeks)	28.1 (15.6–40)	31.9 (15.0–41.1)	0.29
Delivery mode of the immediate previous pregnancy			
Vaginal delivery	9 (64.3)	20 (47.6)	0.28
Cesarean delivery	0 (0.0)	9 (21.4)	0.06
Spontaneous abortion	4 (28.6)	3 (7.1)	0.05
Dilatation and curettage	1 (7.1)	10 (23.8)	0.17
Timing of RPOC diagnosis (days)	23 (5–51)	36 (5–69)	0.0479
Ultrasonographic findings			
Grayscale ultrasonographic finding			
RPOC long-axis length (cm)	44.0 (15–72)	23.5 (3–58)	0.0103
RPOC short-axis length (cm)	16.5 (10.0–40.0)	17.0 (1.0–37.0)	0.28
Color Doppler ultrasonographic finding			
RPOC hypervascularity	9 (64.3)	12 (28.6)	0.0168
Blood test			
Serum hCG concentration (mIU/mL)	19 (0–599)	12 (0–820)	0.42
Serum Hb concentration (g/dL)	11.7 (6.4–13.0)	11.7 (3.8–13.6)	0.05

The effects of the potential predictors of RPOC with severe PPH are shown in Table 2. According to crude analyses, second-trimester miscarriage (OR, 5.28; 95% CI, 1.18-23.71), TIME (OR, 4.06; 95% CI, 1.15-16.88), long-axis SIZE (OR, 12.67; 95% CI, 3.07-61.83), and RPOC hypervascularity (OR, 4.50; 95% CI, 1.29-17.40) were significantly associated with an increased risk of RPOC with severe PPH. Furthermore, according to the multivariable analysis incorporating information from chosen obstetric histories (Model A), neither second-trimester miscarriage (OR, 4.42; 95% CI, 0.92-22.68) nor TIME (OR, 3.54; 95% CI, 0.95-15.30) was a significant predictor of RPOC with severe PPH.

**Table 2:** Crude and multivariable ORs for RPOC with severe postpartum hemorrhage Model A, a combination of second-trimester miscarriage and TIME; Model B, a combination of Model A and long-axis SIZE; Model C, a combination of Model B and RPOC hypervascularity. CI: Confidence interval; OR: Odds ratio; RPOC: Retained products of conception; SIZE: RPOC long-axis length; TIME: Time at which there was clinical suspicion of RPOC after the previous pregnancy.

Parameters	Crude OR (95% CI)	Multivariable OR (95% CI)		
		Model A	Model B	Model C
Second-trimester miscarriage	5.28 (1.18–23.71)	4.42 (0.92–22.68)	5.30 (0.87–34.60)	5.19 (0.75–38.85)
TIME	4.06 (1.15–16.88)	3.54 (0.95–15.30)	2.13 (0.46–10.32)	2.16 (0.45–11.08)
Long-axis SIZE	12.67 (3.07–61.83)		11.12 (2.34–63.39)	10.38 (2.06–63.86)
RPOC hypervascularity	4.50 (1.29–17.40)			3.29 (0.73–16.46)

With further adjustment for grayscale ultrasonographic findings (Model B), long-axis SIZE was associated with a high risk of RPOC with severe PPH (OR, 11.12; 95% CI, 2.34-63.39). Upon further adjustment, long-axis SIZE was the only significant predictor of RPOC with severe PPH (OR, 10.38; 95% CI, 2.06-63.86) independent of second-trimester miscarriage, TIME, and RPOC hypervascularity. These models were subjected to multivariate logistic regression analysis to evaluate the diagnostic value of the combination of the three models. The probability of the models were as follows:

Model A probability = 1/(1 + exp [−0.72 − 0.74 × second-trimester miscarriage − 0.63 × TIME]);

Model B probability = 1/(1 + exp [−0.38 − 0.68 × second-trimester miscarriage − 0.52 × TIME − 0.59 × long-axis SIZE]);

Model C probability = 1/(1 + exp [−0.087 − 0.82 × second-trimester miscarriage − 0.39 × TIME − 1.17 × long-axis SIZE − 0.59 × RPOC hypervascularity]).

**Figure 2:**
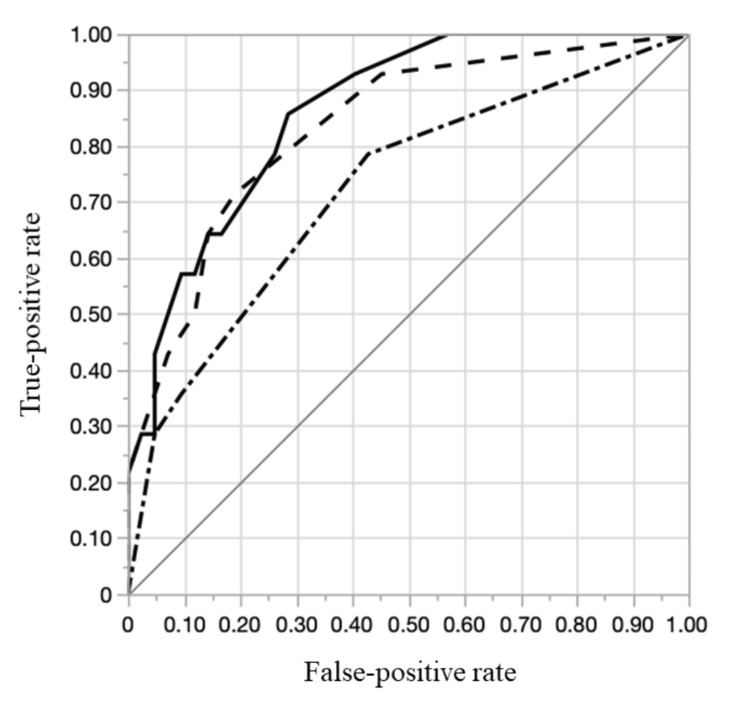
Receiver operating characteristic curves of the multivariable logistic regression analysis The solid line represents the multivariable logistic regression of Model C (c-statistic: 0.863; 95% confidence interval [CI], 0.731–0.936); the dotted-dashed line represents that of Model A (c-statistic: 0.723; 95% CI, 0.551–0.847); and the dotted line represents that of Model B (c-statistic: 0.834; 95% CI, 0.677–0.923).

A receiver operating characteristic curve was used to demonstrate the ability of the statistical models to discriminate between patients with PPH versus those without severe PPH (Figure 2 and Table 3). The c-statistic was higher in Model C (0.863; 95% CI, 0.731-0.936) than in Model A (0.723; 95% CI, 0.551-0.847) or Model B (0.834; 95% CI, 0.677-0.923). The accuracy of RPOC with severe PPH based on Model C had a sensitivity of 85.7% (95% CI, 63.9-95.8), specificity of 71.4% (95% CI, 64.2-74.8), positive predictive value (PPV) of 50.0% (95% CI, 37.3-55.9), and negative predictive value (NPV) of 93.8% (95% CI, 84.2-98.2). Model A had a sensitivity of 78.6% (95% CI, 56.0-92.1), specificity of 57.1% (95% CI, 49.6-61.6), PPV of 37.9% (95% CI, 27.0-44.4), and NPV of 86.9% (95% CI, 77.2-95.9). Model B had a sensitivity of 71.4% (95% CI, 49.5-86.8), specificity of 81.0% (95% CI, 73.7-86.1), PPV of 55.6% (95% CI, 38.5-67.5), and NPV of 89.5% (95% CI, 81.4-95.2).

**Table 3:** Receiver operating characteristics of RPOC with severe postpartum hemorrhage Model A, a combination of second-trimester miscarriage and TIME; Model B, a combination of Model A and long-axis SIZE; Model C, a combination of Model B and RPOC hypervascularity. CI: Confidence interval; NPV: Negative predictive value; PPV: Positive predictive value; RPOC: Retained products of conception; TIME: Time at which there was clinical suspicion of RPOC after the previous pregnancy; SIZE: RPOC long-axis length.

	c-statistic (95% CI)	Sensitivity, % (95% CI)	Specificity, % (95% CI)	PPV, % (95% CI)	NPV, % (95% CI)
Model A	0.723 (0.551–0.847)	78.6 (56.0–92.1)	57.1 (49.6–61.6)	37.9 (27.0–44.4)	86.9 (77.2–95.9)
Model B	0.834 (0.677–0.923)	71.4 (49.5–86.8)	81.0 (73.7–86.1)	55.6 (38.5–67.5)	89.5 (81.4–95.2)
Model C	0.863 (0.731–0.936)	85.7 (63.9–95.8)	71.4 (64.2–74.8)	50.0 (37.3–55.9)	93.8 (84.2–98.2)

## Discussion

This study showed three novel significant findings as follows: (1) The combination of four risk factors (second-trimester miscarriage, TIME, long-axis SIZE, and RPOC hypervascularity) improved the predictive diagnostic accuracy of RPOC with severe PPH. (2) The cut-off value for TIME was <27 days, while it was >4.4 cm for long-axis SIZE. (3) Long-axis SIZE was an independent risk factor for RPOC with severe PPH.

The prediction model for RPOC with severe PPH identified the four factors of second-trimester miscarriage, TIME, long-axis SIZE, and RPOC hypervascularity. Furthermore, long-axis SIZE was an independent risk factor with a cut-off value of >4.4 cm. These findings are supported by recent reports. First, an RPOC length > 4.0 cm and RPOC hypervascularity were independent risk factors for interventions [11]. Second, the ultrasonographic hypervascularity of RPOC was a predictor of surgical procedures [21]. Additionally, the long-axis length in RPOC aids the prediction of severe PPH requiring invasive treatment, with a cut-off value of >4.4 cm [22].

This study further improved the accuracy of the prediction model for RPOC with severe PPH by incorporating two additional parameters of second-trimester miscarriage and TIME. Several previous reports support these findings, indicating that second-trimester miscarriage is responsible for approximately 40% of RPOC [23], and the risk of RPOC after second-trimester miscarriage increases [24]. Additionally, the occurrence of RPOC with profuse bleeding within 27 days postpartum is a risk factor that should be considered in clinical management. This outcome illustrates the crucial importance of early diagnosis in clinical management. Therefore, postpartum management incorporating color Doppler is considered necessary for RPOC.

We found four risk factors (second-trimester miscarriage, TIME, SIZE, and RPOC hypervascularity) in the predictive detection of severe PPH. We used a logistic regression analysis to analyze the effect of these four risk factors on severe PPH. We chose to use a logistic regression model because it is a common method for analyzing the degree of the effect of multiple independent variables on a single dependent categorical outcome. Logistic regression models were used to assess the predictors of RPOC with severe PPH as follows. Finally, the accuracy of the prediction models was evaluated using receiver operating characteristic curves. We made statistical inferences about predicting RPOC with severe PPH from multiple risk factors using a logistic regression analysis. However, there needs to be caution regarding incorrect inferences due to multicollinearity for various risk factors.

Model C (c-statistic: 0.863) improved the predictive diagnostic accuracy of RPOC with severe PPH. Furthermore, this prediction model for RPOC with severe PPH was considered suitable as a clinical screening tool because it showed a high NPV. Therefore, this prediction model for RPOC with severe PPH can effectively identify cases that may be managed conservatively [25]. In addition, avoiding invasive surgical procedures, such as hysterectomy, hysteroscopic transcervical resection, and D&C, is clinically beneficial for patients [13,26].

There have been various reports on the efficacy of conservative management for RPOC [10-12]. However, concomitant drug therapy is crucial for ensuring the reliability of this elective management approach. As a potential combination drug therapy, management using Kampo products, uterotonic agents [27,28], and antibacterial agents [29] may prove beneficial. Unfortunately, Kampo products related to RPOC are limited. Although we have clinical experience with Tokakujokito (Tao He Cheng Qi-Tang: 61), which is a formula obtained from Tsumura & Co., Ltd. (Tsumura; Tokyo, Japan) that has been used for an extended period with few reported side effects, scientific evidence to support its clinical use is lacking. We will continue to accumulate cases and verify the effects of combination therapy in the future.

This study showed no significant intergroup difference in conception by ART. The RPOC with severe PPH group tended to have higher hCG concentrations and lower Hb concentrations than the RPOC without severe PPH group, but these differences were not significant. In contrast, a recent study reported that ART and low serum Hb concentrations were potential predictive factors for RPOC with massive hemorrhaging [30]. In measuring serum biomarkers, the timing of blood sampling may alter the significance of the test results. Therefore, further research on the extent of these changes would aid in decision-making for conservative management or invasive surgical management.

There are five limitations to this study. First, we used a retrospective design, which may have introduced potential bias in the decision-making regarding blood transfusion therapy. However, this limitation is inherent because treatment decisions are based on multiple factors. Second, the inclusion criteria relied on clinically diagnosed RPOC using ultrasound and magnetic resonance imaging. The pathological diagnosis of RPOC was not possible in all cases, particularly in those of conservative management because a pathological diagnosis cannot be made when the uterine contents are naturally expelled. This is an inherent limitation of this study. Third, the predictive model can be used to determine hospitalization management for severe PPH. However, this model does not provide clear guidance on the effectiveness of adjuvant treatment in combination with elective management. Fourth, different observers, timing, and models of ultrasound examinations may have affected the study results. Fifth, 13 cases of RPOC without severe PPH were managed expectantly. Therefore, we did not perform any surgical procedures, and there were no pathological diagnoses of uterine contents. This dilemma is a possible topic for future investigation.

## Conclusions

In conclusion, the combined use of a specific obstetric history (second-trimester miscarriage and TIME) and ultrasonographic findings (long-axis SIZE and RPOC hypervascularity) can improve the predictive accuracy of RPOC with severe PPH. Additionally, the predictive accuracy of RPOC with severe PPH improved with an increasing number of combinations of the four risk factors identified in our study; all combinations had the highest predictive accuracy of RPOC with severe PPH. Therefore, this prediction model may serve as a useful clinical screening tool for RPOC with severe PPH. Furthermore, this model may greatly affect the determination of appropriate management strategies for RPOC.
